# Underwater audiogram of the grey seal (*Halichoerus grypus*)

**DOI:** 10.1242/bio.062059

**Published:** 2025-08-19

**Authors:** Andreas Ruser, Kirstin Anderson Hansen, Magnus Wahlberg, Tobias Schaffeld, Ursula Siebert

**Affiliations:** ^1^ Institute for Terrestrial and Aquatic Wildlife Research, University of Veterinary Medicine Hannover, Foundation, 25761 Büsum, Germany; ^2^ Marine Biological Research Center, Department of Biology, University of Southern Denmark, 5300 Kerteminde, Denmark

**Keywords:** Pinniped, Underwater hearing, Psychophysics, Grey seal, Audiogram

## Abstract

The hearing sensitivity of two grey seals (*Halichoerus grypus*) was measured using a psychophysical staircase method from 0.125 to 64 kHz. Both animals had best hearing sensitivity at 4 kHz (43-44 dB re 1 µPa). Below 4 kHz, hearing thresholds differed. These first underwater audiograms using psychophysical methods demonstrate that high-frequency hearing in grey seals is comparable to other phocids. However, they show better sensitivity below 8 kHz, indicating that low-frequency hearing may play a more important role in this species, potentially increasing its vulnerability to anthropogenic noise. Notably, hearing thresholds at low frequencies were lower than predicted by the standard audiogram used to generate phocid frequency weighting function, which is currently used to assess the impact of anthropogenic sound on marine mammal hearing. These functions may not adequately represent grey seal sensitivity at low frequencies. Our results may, if corroborated by further audiograms, motivate modifications of the phocid standard audiogram.

## Teaser

**Summary:** The hearing sensitivity of two grey seals was measured using a psychophysical staircase method from 0.125 to 64 kHz. Notably, hearing thresholds at low frequencies were lower than predicted by the standard audiogram used to generate phocid frequency weighting function.

## INTRODUCTION

The evolutionary adaptations of hearing in seals are closely linked to their specific habitats and lifestyles (Berta, 2018). Fossil evidence indicates significant changes in auditory structures as pinnipeds adapted to aquatic environments, resulting in a diversity in hearing abilities across different families of pinnipeds. Early pinniped fossils, such as *Enaliarctos mealsi*, displayed both terrestrial and aquatic adaptations, suggesting a gradual shift in sensory and structural capabilities to support underwater hearing (Berta et al., 1989; Møhl, 1968; Repenning and Harrison, 1972). These anatomical changes, particularly in middle ear morphology, reflect adaptations that likely enhanced underwater sound transmission, a critical feature for aquatic life (Duarte et al., 2021; Fleischer, 2013; Taszus et al., 2023).

The acute hearing abilities of pinnipeds make them vulnerable to anthropogenic noise. Seals hear well both in air and underwater, but their hearing abilities seem to be adapted in different ways to the underwater environment (Reichmuth et al., 2013), where they spend most of their time hunting and navigating. The effects of underwater noise on marine wildlife are currently a large concern globally (Duarte et al., 2021; Hildebrand, 2009). Primary contributors to the underwater soundscape are vessels, sonars, acoustic deterrent devices, construction work, explosions, and seismic surveys. These sources contribute to an escalating ‘anthrophony’ that has intensified over recent decades due to industrial expansion. Levels are expected to rise further with ongoing coastal as well as offshore developments. Seals can significantly change their behaviour as a result of noise exposure (Martin et al., 2022; Mikkelsen et al., 2019; Prawirasasra et al., 2022), and it is conceivable that noise may challenge them by masking sounds from conspecifics, prey and predators, as well as compromising navigation by passive listening.

Given the prevalence of low-frequency sounds in many sources of anthropogenic noise, it is important to investigate the low-frequency hearing sensitivity of phocid seals to assess their vulnerability to anthropogenic noise. Phocid seals have anatomical adaptations that may favour low-frequency underwater hearing (Hemilä et al., 1995). They also have blood-filled cavernous sinuses to enhance sensitivity of underwater sounds (Møhl, 1967; Smodlaka et al., 2019).

Fine-scale noise effects on marine mammal hearing and behaviour have been documented through a combination of psychophysical, physiological, and behavioural studies of trained animals, as well as field observations of wild animals (Mikkelsen et al., 2019; Southall et al., 2019; Tougaard et al., 2015; Wisniewska et al., 2018). In Northern European waters, substantial knowledge has accumulated on the impacts of noise on particularly harbour porpoises and harbour seals (Kastelein et al., 2016; Mikkelsen et al., 2019; Reichmuth et al., 2013; Tougaard et al., 2015; Wisniewska et al., 2018).

However, a knowledge gap remains for other phocids presumed to have excellent underwater hearing abilities, such as the grey seal (*Halichoerus grypus*, Fabricius, 1791). Grey seals, one of the most common seal species in the North Atlantic, are dominant top predators in many coastal and offshore ecosystems. These ecosystems are currently undergoing a rapid change due to factors such as new fishing regulations, modifications in oil and gas production and the expansion of offshore windfarms. Grey seals are central-place foragers, preying on a variety of taxa (Huon et al., 2021; Riedmann, 1990). They spend most of their time foraging within 10 to 50 km of the coast (Huon et al., 2015; Jones et al., 2015), but foraging trips with distances up to more than 1000 km have been observed, highlighting their adaptability to different ecological conditions (Huon et al., 2021).

In response to the growing impact of anthropogenic noise on marine life, management strategies increasingly rely on frequency weighting functions to estimate perceived loudness and assess the risk potential for auditory impacts. These functions are used to predict which frequencies are most likely to induce temporary threshold shifts or other auditory effects, thereby informing regulations on permissible noise levels and guiding mitigation measures (National Marine Fisheries Service, 2024; Southall et al., 2019, 2021). However, current weighting functions, such as those developed for phocid carnivores in water (PCW), are based on aggregated data from multiple species and may not fully account for species-specific sensitivities. Given that grey seals have not been evaluated behaviorally and are thus not accounted for in current regulations, accurately capturing their hearing thresholds is critical to effectively assess and mitigate noise impacts on this species.

In this study, we measure underwater audiograms of two male grey seals using a psychophysical technique. Our data allow the first assessment of underwater hearing thresholds in grey seals and provide insights into their sensitivity across frequencies relevant to anthropogenic noise impacts. Our findings contribute to understanding the effects of noise exposure for grey seals and emphasize the importance of developing frequency weighting functions that accurately reflect their low-frequency hearing sensitivity.

## RESULTS AND DISCUSSION

For both animals' audiograms, best hearing sensitivity was at 4 kHz, with a hearing threshold of 43-44 dB re 1 µPa (Table 1). Below 4 kHz, the hearing thresholds of grey seal referred to as ‘Hg_1’ were 6.0 to 13.3 dB lower than those of grey seal referred to as ‘Hg_2’. For frequencies between 4 and 40 kHz, the hearing thresholds for both seals were within 1 dB, except at 8 kHz with an 8.8 dB lower threshold for Hg_2. Above 40 kHz, the differences in thresholds were below 5 dB.

**Table 1.** Underwater hearing thresholds measured for two grey seals using psychophysical method and the power spectral density of the background noise

	Hg_1					Hg_2					Noise
Freq.	Thres.	SD_wm_	*N*	Rev.	FA	Thres.	SD_wm_	*N*	Rev.	FA	PSD
(kHz)	(dB re 1 µPa)				(%)	(dB re 1 µPa)				(%)	(dB re 1 µPa²Hz^−1^)
0.125	68.1	0.9	9	58	10.7	77.4	0.9	9	68	7.6	65.7±6.6
0.25	56.3	0.9	10	62	13.0	69.6	0.8	10	66	9.9	57.1±7.3
0.5	51.9	1.0	9	58	13.6	57.9	0.8	10	66	8.7	47.1±10.1
1	46.4	0.7	8	56	9.5	58.4	0.7	9	62	7.1	45.8±6.0
2	50.5	0.9	10	48	13.5	58.0	1.0	8	52	3.4	40.2±6.5
4	42.9	0.9	8	66	15.3	43.8	0.8	9	66	9.8	38.4±5.4
8	64.8	0.9	9	62	11.7	56.0	1.1	10	70	13.4	37.6±5.5
16	63.6	1.0	10	64	10.6	64.2	1.0	8	58	5.4	33.9±6.6
32	58.4	1.0	7	50	12.5	58.1	1.0	8	52	10.7	28.8±4.7
40	68.0	1.1	9	48	4.2	67.3	1.1	8	52	8.1	29.2±4.0
51	66.1	1.0	7	38	7.9	70.1	1.4	10	58	5.3	29.0±3.6
64	103.0	1.0	10	64	7.0	98.3	1.0	9	48	7.5	28.7±2.7

FA, mean false alarm rate; Freq., frequency; *N*, number of valid sessions; PSD, power spectral density (±s.d.); Rev., total number of reversals; SD_wm_, standard deviation of weighted mean; Thres., weighted mean of sessions per frequency.

The number of reversals per session ranged from four to 12. The average number of reversals per session (over all 214 sessions) was 6.5±2.0 reversals. The median reversal per frequency and animal was 60±8 reversals.

The hearing thresholds of the two grey seals were similar for many frequencies, with some interesting low- and mid-frequency deviations. Studies in other pinnipeds have documented comparable individual variability in hearing thresholds. Individual differences of up to 8 dB were observed in spotted seals (Sills et al., 2014), 6 dB in ringed seals in the frequency range of 0.1 to 25.6 kHz (Sills et al., 2015), and up to 7 dB at 3.2 kHz in bearded seals. Similar and even larger individual differences were observed in California sea lions (*Zalophus californianus*), which Kastak and Schusterman (1998) attributed to age-related differences. Both grey seals in our study lived in a controlled zoo environment and had similar life histories with only a 3-year age difference, and they were born and raised in the same facility, with identical training and comprehensive medical documentation. The difference in the grey seals' low-frequency thresholds therefore likely reflects natural interindividual differences rather than extrinsic environmental factors (e.g. caused by previous noise exposure). Even though more data are needed to explore the full variation in grey seal hearing sensitivity, our results show that individual variability may be large in this species, and potentially also in other phocids.

**Fig. 1.**
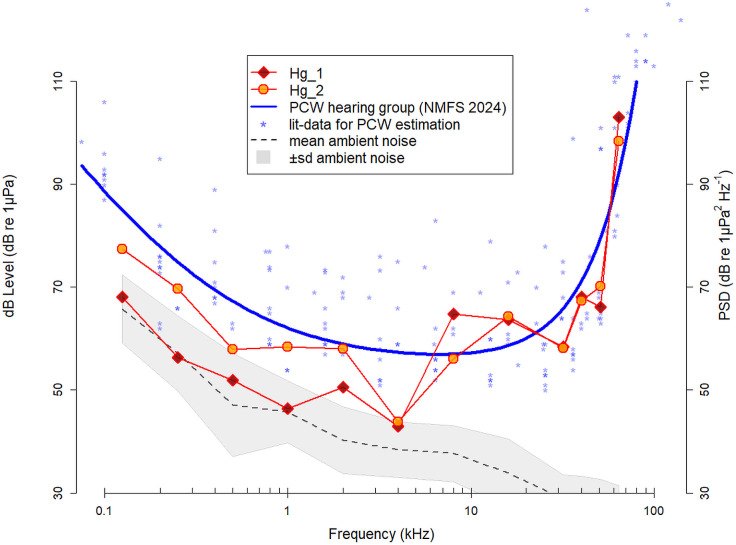
**Underwater audiograms for the two grey seals.** Hearing thresholds for Hg_1 (dark-red filled red diamonds) and Hg_2 (orange filled red circles) and for comparison the estimated group audiogram taken from the National Marine Fisheries Service (2024) for phocid carnivores in water (PCW; blue line). Threshold values of 14 phocids, derived by psychophysical methods, used for the estimated PCW audiogram (Table A.1-1 in National Marine Fisheries Service, 2024), are shown as blue asterisks (seven thresholds greater than 125 dB are not shown). The grey dashed line indicates the median of the ambient noise level (PSD) with s.d. (grey filled area).

Grey seal hearing thresholds above 4 kHz are very similar to those of other seal species, including harbour seals (Kastelein et al., 2018; Reichmuth et al., 2013), ringed seals (Sills et al., 2015), spotted or Largha seals (Sills et al., 2014), and bearded seals (Sills et al., 2020). The grey seal Hg_2 showed comparable sensitivity to frequencies below 4 kHz to harbour seals measured in Kastelein et al. (2009, 2018) [note: except for the harbour seals' low threshold at 200 Hz in Kastelein et al. (2009), not confirmed in Kastelein et al. (2018)]; but, compared to the results on ‘Sprouts’ in Reichmuth et al. (2013) in same frequency range (but not at the same frequencies), they are 7-20 dB lower. An exception are the two low hearing thresholds of 43-44 dB of grey seals at 4 kHz, which are 16 dB lower than those of harbour seals (Kastelein et al., 2009) and 7-9 dB lower than those of bearded seals (Sills et al., 2020). On the other hand, below 4 kHz, Hg_1 has hearing thresholds 6 to 13 dB lower than Hg_2, and significantly lower than those of harbour seals and bearded seals. The thresholds of Hg_1 are close to the spectral ambient noise levels (Table 1, Fig. 1), which at first glance could indicate them being masked. However, this may not be the case, as any time-frequency structure in the ambient noise may lead to co-modulation masking release, a mechanism known from other marine mammals (Branstetter and Finneran, 2008). An influence of the perception of particle motion on our results could be neglected (more details are in the ‘Hearing studies in a concrete pool’ section). Since the results are unusually low, confirmation of the hearing thresholds would be highly desirable.

Seals rely on their sense of hearing to perceive acoustic signals for mating, socializing, and defending their territories (Pozo Galván et al., 2024). Seals exhibit species-specific differences in vocalization frequency, signal frequency, and the context of social interactions. Grey seals are particularly dependent on low-frequency vocalizations (Asselin et al., 1993; Hanggi and Schusterman, 1994; Pozo Galván et al., 2024; Stirling and Thomas, 2003; Van Parijs et al., 2000). Grey seals vocalize primarily in a frequency range between 100 Hz and 3 kHz. Males emit rhythmic, low-frequency calls, particularly during the breeding season, to mark territories and attract mates (Asselin et al., 1993; Pozo Galván et al., 2024; Van Parijs et al., 2000). Detecting other important acoustic cues in their environment may also be crucial. For example, seals likely use their hearing to forage, where sounds emitted by fish could be used to detect and locate potential prey (Hanke and Reichmuth, 2022).

Anthropogenic noise exposure can be harmful to marine mammals, and therefore government agencies often issue guidelines and regulation for noisy activities such as pile driving (BMU, 2013; Verfuss et al., 2016, 2019; Wagenknecht, 2021) and seismic exploration. So-called weighting functions can improve the prediction of the effects of anthropogenic noise on marine mammals (Houser et al., 2017). The hearing thresholds of grey seals are below the thresholds of the standard audiogram used to generate the frequency weighting function for phocids (National Marine Fisheries Service, 2024), based on audiograms derived from aggregated hearing data from 14 phocids of seven different species (Table A.1-1 in National Marine Fisheries Service, 2024), but not including grey seals. The results of behavioural studies on the hearing sensitivity of a species in different studies, however, show considerable variation (Cunningham and Reichmuth, 2016; Kastak and Schusterman, 1999; Kastelein et al., 2009; Reichmuth et al., 2013; Sills et al., 2014, 2015, 2020, 2021; Terhune, 1988; Terhune and Ronald, 1972). For example, five audiograms of harbour seals were used to determine the phocid weighting function. Three of these studies include data at low frequencies, where thresholds vary by more than 15 dB. Likewise, the threshold differences between species are large: when comparing the most sensitive species (harbour seals and bearded seals; Kastelein et al., 2009; Sills et al., 2020) and the least sensitive ones (Hawaiian monk seals; Sills et al., 2021), the difference is as high as 15-25 dB for frequencies below 8 kHz. For grey seals, our findings indicate that the PCW group audiogram underestimates their sensitivity at lower frequencies (Fig. 1). Increasing noisy anthropogenic activities (Halpern et al., 2008, 2015) underscores the importance of assessing the impact of low-frequency human sound on marine mammals. Applying a generalized sensitivity function for phocid hearing when assessing the risks of low-frequency sound exposure to grey seals could lead to an underestimation of potential hearing impacts, particularly in individuals with hearing thresholds such as Hg_1. To improve the efficiency of mitigation measures, it may be pertinent to derive separate weighting functions for each phocid species, once such data are becoming available.

## MATERIALS AND METHODS

### Experimental subjects

Underwater hearing thresholds were investigated from 2018 to 2021 in two male grey seals, born in captivity in 2014 (referred to as Hg_1) and 2017 (referred to as Hg_2). The seals arrived at 2 months of age from Kolmården Zoo, Sweden, to the Marine Biological Research Centre (University of Southern Denmark) in Kerteminde, Denmark. Both seals were healthy and had not been exposed to ototoxic medications before or during the experiments. Their weight varied between 100 and 150 kg due to seasonal variation. They were housed in an outdoor enclosure consisting of three round saltwater pools of 5 to 7 m diameter and 1.7 m depth and surrounded by wooden decks. Experiments were conducted in an underground 7 m diameter, 1.7 m deep concrete, tarp-lined pool. Water from the neighbouring harbour was pumped to the pools, so that the seals always experienced water with ambient temperature (ranging from 0 to 20°C) and salinity (10-20 ppm), as well as natural ambient air temperatures. To reduce the risk of auditory masking during psychophysical experiments, all pumps were shut off during testing, and testing was not made when it was raining. Since their arrival, both seals received extensive training in classical and operant conditioning and were involved in separate psychophysical, experiments for pinger signals. Both seals were successfully trained for a psychophysical GO/NO-GO testing paradigm with an 80% hit rate for suprathreshold sessions. Their food consisted of herring (*Clupea harengus*, Linnaeus, 1758), sprat (*Sprattus sprattus*, Girgensohn, 1846) and capelin (*Mallotus villosus*, Müller, 1776).

### Training and equipment

**Fig. 2.**
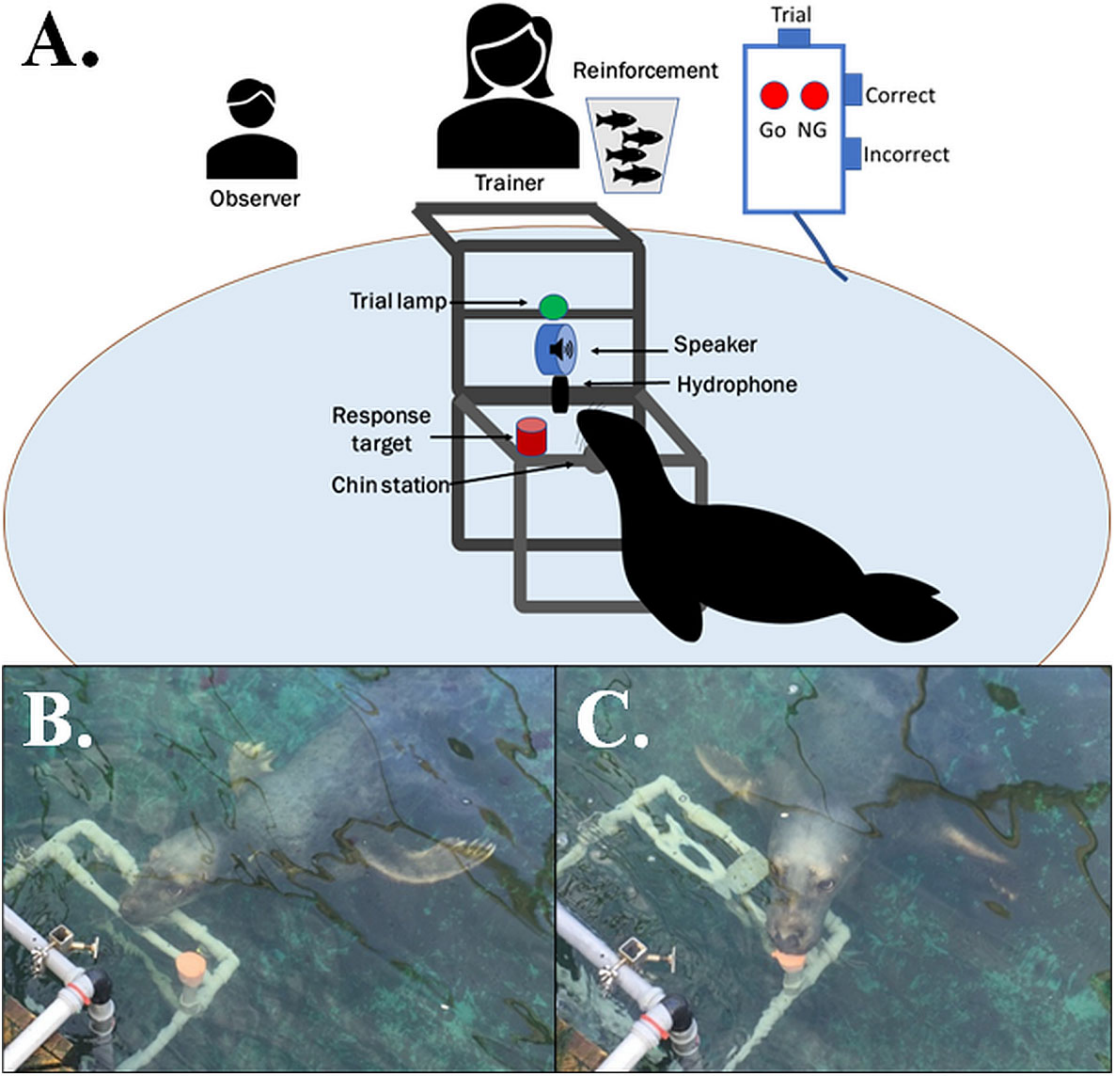
**Schematic of underwater setup for psychophysical research, including the trial console and the position of the seal at station.** (A) The trial start button initiated either a ‘signal-present’ or a ‘signal-absent’ trial. At the completion of the trial, either the GO or NOGO lamp turned on, indicating to the trainer which trial type had been presented to the seal. The trainer would then either confirm a correct response by pressing the ‘correct’ button, which also presented the secondary reinforcer, or the ‘incorrect’ button, recalling the seal to the trainer at the surface. (B) Correct start position at the chin station, for each trial. For a correct rejection, the seal was required to stay stationed on the chin station until the end of the trial (4 s). (C) For a hit, the seal left the station upon presentation of the sound stimulus and touched the response target on the left of the chin station with his nose.

Seal training was based on positive reinforcement, and hearing was tested using a GO/NO-GO testing paradigm (Gescheider, 1997). The subject was trained to swim voluntarily to an underwater station 70 cm below the water surface, where he rested his head on a chin station mounted on a rig made from PVC pipes. On an additional PVC rig, mechanically separated from the chin station, a trial light and two transducers (for specifications, see below) were mounted. The additional rig was attached to the pool edge, directly in front and at distance of 80 cm from the chin station (Fig. 2A). At the start of each trial, the trial light was turned on and remained on for the duration of the trial (4 s).

There were four possible responses from the seal for the GO/NO-GO testing paradigm. For the trials where a signal was presented, the seal could respond with a hit or a miss. A hit is a correct response and was reinforced with a primary reinforcer (a fish). A miss was an incorrect response and was not reinforced. For trials where no signal was presented, the seal could respond with a correct rejection or a false alarm. A correct rejection was a correct response and was reinforced with a primary reinforcer (a fish). A false alarm was an incorrect response and was not reinforced.

For a hit, the seal left the station upon presentation of the sound stimulus and touched the response target on the left of the chin station with his nose (Fig. 2B). This behaviour resulted in the trainer pressing the ‘correct’ button on the console, causing the emission of an underwater conditioned reinforcer (a so-called bridge; a 0.5 s 1-8 kHz buzz signal), followed by an unconditioned reinforcer (a fish of the species capelin), given by the trainer. For a miss, the seal stayed at the chin station for the duration of the trial for a signal present trial. At the completion of the trial, the trainer pressed the ‘incorrect’ button, and the seal was recalled to the surface, where no reinforcement was given, after which it was sent down to the chin station for the next trial. For a correct rejection, the seal was required to stay stationed on the chin station until the end of the trial (4 s) (Fig. 2C). The trainer pressed the ‘correct’ trial button, resulting in the conditioned reinforcer (bridge), followed by an unconditioned reinforcer (a capelin) given by the trainer. If the seal did not respond within the 4 s response time, his response was considered incorrect. He was then recalled to the surface, where no reinforcement was given, and sent down to the chin station for the next trial after the trainer had pressed the ‘incorrect’ button. For a false alarm, the seal left the station and touched the response target during the trial. The trainer pressed the ‘incorrect’ button, and the seal was recalled to the surface, where no reinforcement was given, and sent down to the chin station for the next trial.

The seals' diets were not constrained for research purposes, with both seals receiving one-third to one-half of their daily diet during experimental sessions. They received their full daily diet ration irrespectively of performance during experiments. The seals were maintained at healthy body weights, according to age and season, for the duration of the experiments. Experimental sessions were made at the same time of the day, about an hour after a first brief morning feed. In this way, we ensured that the seals had received approximately the same amount of fish prior to each experimental session so as to not affect their motivation during testing.

Experimentation was made under permit number 2020/01 from the Animal Welfare Committee at University of Southern Denmark, authorized by the Danish Animal Experiment Inspectorate.

### Testing parameters

A staircase method with 3 dB decrements and increments was used during testing. Each session contained 30-45 trials, including four warm-up trials and four cooldown trials, where the signal level was easily detectable by the seal, at least 25 dB above threshold. Besides stimulus present trials, we also included 40% of stimulus absent (catch) trials. To avoid any predictability in the pattern of stimulus present/absent trial types, 12 separate pseudorandom sequences were created following Gellerman (1933), out of which one sequence was chosen randomly for each session.

To avoid secondary cues, the experiment was double-anonymized in that neither the subject nor the trainer had prior knowledge of trial type. The trainer sat at the edge of the pool, above the test set-up. A small handheld console was controlled by the trainer (Fig. 2A). It contained a trial start button, a correct response button, which also triggered the underwater conditioned reinforcer, and an incorrect response button. Once the trainer hit the trial start button, the trial light turned on, and a pre-programmed randomizing sequence initiated either a signal-present or a signal-absent trial. A lamp on the console indicated trial type upon completion of the trial. Both trial types were initiated in the same way, with the only difference being that no signal was presented in signal-absent trials. For signal-present trials, the stimulus was presented 1-2 s after the start of the trial. It was not possible for the trainer to hear the stimulus tone during testing, as it was played back underwater at low intensity. The total number of trials, the number of hits, misses, correct rejections and false alarms, as well as the relative intensity of the sound stimulus for each stimulus-present trial was registered and stored automatically on a laptop at the completion of each session. An additional observer was also present to document the seal's response, as well as to note any external activities that might occur during the experiment.

Both seals were tested once per day. Most experiments were run at the same time of the day for consistency in motivation and ambient noise levels. Experiments never had to be postponed due to high ambient noise levels (see below), except during rainfall.

### Testing equipment

The testing paradigm was steered through a custom-made LabView program on a laptop connected with USB to DAQPAD (USB-9162, National Instruments, USA). The output of the DAQPAD was connected to a Basetech AP-2100 or TDA2030A amplifier and to an underwater loudspeaker (University Sound UW-30, Lubell, USA). In trials with a stimulus at frequencies of 16 kHz or beyond, the amplifier was connected to a HS25 (16 kHz) or a HS70 (32 kHz and higher) transducer (both Sonar Products, UK). In the high-frequency trials, the UW-30 underwater loudspeaker was connected via an additional amplifier to the headphone jack of the laptop.

The ambient noise levels were recorded for 5 min prior to each session using either an acoustic datalogger (SoundtrapHF, OceanInstruments, New Zealand, 16 bits, 576 kHz), or a TC4032 (Teledyne Reson, Denmark) hydrophone connected to a TASCAM DR-680MKII recorder (192 kHz, 16 bits). The choice of equipment for ambient noise level measurements was dependent on what equipment was available in the laboratory during different periods of testing. The hydrophone was placed right above the chin station during noise measurements. The hydrophone's frequency-dependent sensitivity was determined using in-air relative calibration with a Grass half-inch microphone for frequencies between 0.125 and 2 kHz, reciprocity calibration (Au and Hastings, 2008) for frequencies between 8 and 64 kHz, and by pistonphone calibration at 250 Hz (with sensitivity interpolation between 250 Hz and 10 kHz; see ‘Calibration of the hydrophone’ section for details). The Soundtrap's sensitivity was determined using relative calibration to the hydrophone. Noise measurements were also made regularly during sessions with the recorder placed next to the transducer to confirm that noise levels were not elevated during trials, as compared to right before trials. The self-noise of the recording systems was measured by placing them inside a sound-proof chamber; the self-noise was lower than the lowest-measured ambient noise levels for the entire frequency band of interest. Noise was analysed using Welsh averaging, with no weighting, and presented as the median average noise level across all used measurements (*N*=31) in a third-octave band centred at each stimulus frequency. Note that not all noise trials were used for analysis, as a significant proportion of them contained high levels of self-noise (mainly caused by the seal swimming around the enclosure creating water movements). Noise variations were reported as the s.d. of each average noise level across all noise measurements.

### Sound stimulus

**Fig. 3.**
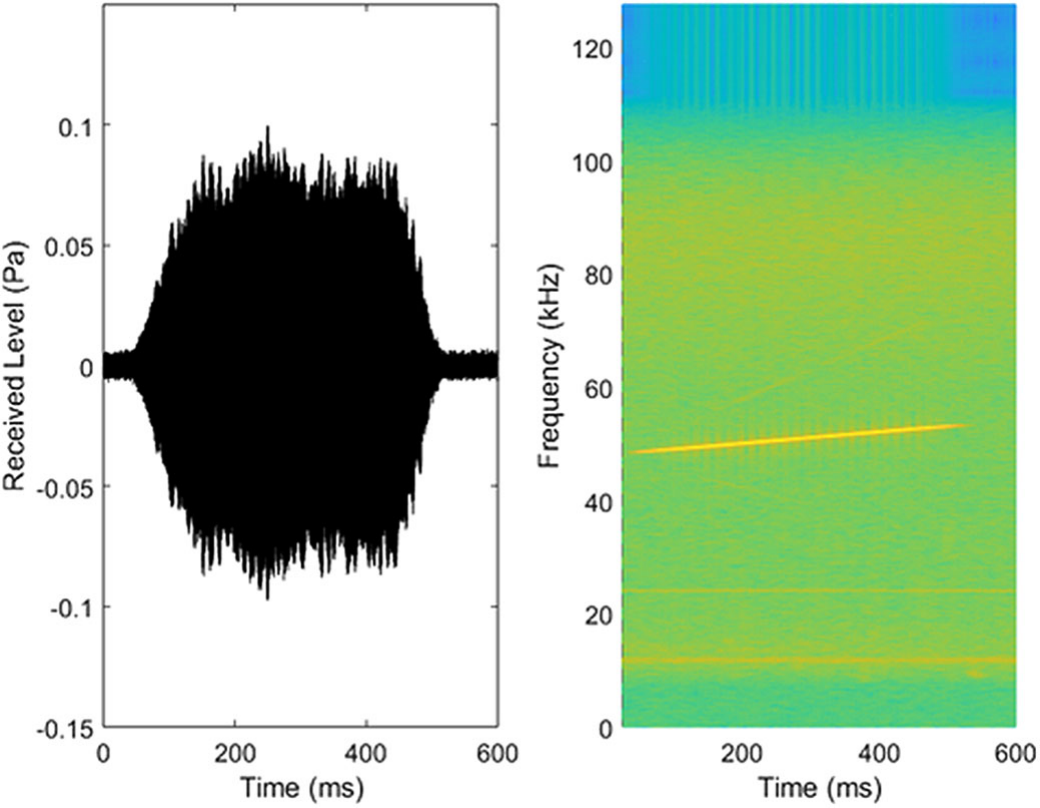
**Oscillogram and spectrogram of 51 kHz stimuli recorded at site of seal.** Left: oscillogram, Right: spectrogram (fast Fourier transform size, 16,384; Hanning window, overlap 90%).

The stimulus was a 0.5 s frequency up-sweep starting 5% below and ending 5% above the tested frequency. The sweep was introduced to suppress interference patterns between direct and reflected paths that could otherwise induce spatial-dependent fluctuations in the received sound level. The signal included a 100 ms ramp-up and 100 ms ramp-down to avoid spectral smearing of stimulus on- and off-set (Fig. 3).

Regular recordings were made of stimulus levels using the same hydrophone and TASCAM recorder used for ambient noise level measurements. The intensity of the generated WAV file was measured using a custom-built Matlab program [root mean square (rms) of the duration of the signal defined by the 95% cumulative energy function; for details see Madsen and Wahlberg (2007)]. The measured level of each stimulus frequency varied by up to ±2 dB between sessions; for each session, the level measured right before the session was used. A recording of each stimulus frequency of 100 emitted stimuli at different locations (±5 cm around seal ear entrance in all Cartesian directions) around the placement of the seal's head resulted in medians varying less than ±3 dB and less than 1 dB variation between signals emitted at the same site (see ‘Spatially varying sound field of stimulus’ section for more information). Finally, an additional hydrophone (B&K8100, Brüel & Kjær, Denmark, fed to a custom-made amplifier, 60 dB, into the TASCAM recorder) was placed right next to the outer ear of the seal during a psychophysical trial. By comparing measurements with and without a seal present at stimulus frequencies of 8, 16 and 64 kHz, it was confirmed that the addition of the seal added a maximum of 2 dB for the stimulus level (see ‘Spatially varying sound field of stimulus’ section). The psychophysical data from the session in which this was tested was not used for hearing threshold determination.

Seals are not only sensitive to sound pressure but also to vibrations. Therefore, the transducer was mechanically uncoupled from the chin rest by being mounted on a PVC rig separated from the PVC rig holding the chin rest. To make sure that near-field particle motion of water was not creating additional cues for the seal during stimulus presentations, we used particle motion measurements made in the same setup and presented in Sørensen et al. (2022). These measurements were made at approximately 60 dB higher sound intensities than the hearing thresholds. The measured particle motion levels were reduced by the same amount of dBs to obtain their magnitude at threshold. At threshold, the particle motion of our stimulus was at least an order of magnitude lower than measured particle motion thresholds for harbour seals by Murphy (2015), for all stimulus frequencies.

### Spatial sound field of hearing test setup, including calibrations of the hydrophone

Initial thresholds were derived based on stimulus recordings made right after every session with a Reson TC4032 hydrophone (#2112050) placed 5 cm above the seal's chin rest. Initially, the nominal hydrophone sensitivity of −170 dB re 1 V/µPa was used to calculate the received level of stimulus. After all psychophysical sessions had been completed, we performed detailed measurements of the sound field using the same TC4032 hydrophone to determine any differences in sound level between the chin rest location and the location of the seals' ears (in the only study made on this topic so far, underwater sound was found to be received in an area around and right below the location of the seal's ear opening; see Møhl and Ronald, 1975). We also investigated if the seal's presence in the setup would have any effect on the sound field during stimulus presentation. The TC4032 hydrophone was oriented vertically (cable upwards) in all measurements. The hydrophone went through detailed sensitivity calibrations, and measured sound levels were adjusted accordingly.

Here, we describe in detail measurements of the sound field of the stimulus as well as the calibrations of the measurement hydrophone and present the correction factors used when reporting both hearing thresholds and background noise levels.

#### Hearing studies in a concrete pool

Conducting hearing studies in a concrete pool has both pros and cons. At higher frequencies, the acoustic field can be hard to control due to interference created by reflections at the pool sides and bottom as well as at the water surface. At low frequencies, excessive particle motion close to the source may confound the sensation of pressure with the sensation of vibration. And finally, noise from water pumps may mask the stimulus. Our careful calibrations of the stimulus sound field as well as the ambient noise in the pool showed that none of these issues are likely to have significantly affected our derived threshold, except for a relatively large variation in the sound field around the seal at 8 kHz. Even though the interval from threshold to ambient noise level was close to or smaller than the critical ratio at some frequencies, this may not indicate that stimulus was masked at threshold: pool noise may include temporal and frequency structure, making the noise less prone to masking the signal than random noise used in critical ratio experiments (see Branstetter et al., 2013), but this has to be corroborated by additional analysis of the recorded noise levels.

#### Spatially varying sound field of stimulus

Measurements of stimuli were made at different locations around the setup to assess spatial variations in the sound field that could potentially affect the perceived sound level by small variations in the seals' location in the setup during trials. We emitted 100 stimuli at all frequencies used for threshold determination, at each location around the setup. The signals were played back through the same transducers (UW30 for 0.125-16 kHz, SP25 for 16 and 32 kHz, and HS70 for 40-64 kHz) as used during psychophysical measurements. Measurements were made with a Reson TC4032 hydrophone (#2112050) held vertically (to avoid any directionality receiver effects) and connected to a TASCAM recorder (recording settings identical to seal stimulus measurements). Sound levels were measured in a custom-made Matlab routine calculating average, maximum and minimum of all levels as dB re 1 µPa rms from a 95% energy window (see Madsen and Wahlberg, 2007).

Measurements were made at the following positions (seal's orientation at station):

1. At chin rest (at location where stimulus level was measured right before trial)
2. 10 cm behind chin rest
3. Right ear, 2 cm left
4. Right ear, 2 cm right
5. Left ear, 2 cm right
6. Left ear, 2 cm left
7. Right ear, 5 cm up
8. Right ear, 10 cm up
9. Right ear, 5 cm towards speaker
10. Right ear, 5 cm away from speaker
11. Left ear, 5 cm up
12. Left ear, 10 cm up
13. Left ear, 5 cm towards speaker
14. Left ear, 5 cm away from speaker

At each location, the sound level varied less than 1 dB. There were relatively small spatial variations in the sound field, except for stimulus frequencies of 4 and 8 kHz where the signal varied up to 8.2 dB compared to the level at the bite plate (Table 2).

**Table 2.** Spatial variations in the sound field around the seal's station during stimulus presentation

Frequency (kHz)	Median variation (dB)	Range (dB)
0.125	−1.8	−4.6−0.1
0.250	−3.5	−4.9 0.4
0.5	−1.3	−3.9 0.1
1	−1.9	−2.3−0.1
2	1.2	0.5 2.6
4	−5.2	−7.1−3.6
8	−6.3	−8.2−1.3
16	−2.0	−3.7 0.0
32	0.8	0.0 1.8
40	2.3	−1.1 3.0
51	1.2	−0.5 3.4
64	1.4	−1.0 2.3

#### Stimulus level at location of seal ear, with and without seal on station

To investigate how the seal head and body could potentially change the sound field by its presence, stimulus measurements were made with and without seal present. A B&K 8100 hydrophone was mounted on the setup close to the site of the seal's ear when it was at station. Stimulus measurements were made at 8, 32 and 64 kHz with and without the presence of the seal, with five to ten measurements for each frequency. Measurements were made in dB re 1 µPa rms over a 95% energy window (Madsen and Wahlberg, 2007). The seal's presence in the setup had a negligible effect on the sound field at all measured frequencies. At 8 kHz, the relative change in stimulus intensity when the seal was present at recording location 2 cm left at the right ear was less than 1 dB from the level when the seal was absent; for 16 kHz, the difference was +2 dB and for 64 kHz it was +1 dB.

#### Calibration of the hydrophone

We determined the sensitivity of the hydrophone (Reson TC4032, #2112050) used throughout the psychophysical sessions for stimulus measurements and ambient noise recordings. We determined the hydrophone sensitivity for all relevant frequencies, which required us to use a range of methods, as each method is not feasible for the entire frequency range. We used both pistonphone, relative and reciprocity calibration (Au and Hastings, 2008). The nominal sensitivity given by the manufacturer for this hydrophone was −170 dB re V/1 µPa.

##### Pistonphone calibration

We attached a B&K pistonphone 4223 with a custom-made coupler. With the hydrophone (Reson TC4032, #2112050) inside the coupler, the pistonphone emitted a signal at 250 Hz and with a received level of 153 dB re 1 µPa rms. The hydrophone output measured with an oscilloscope was 180 mV rms. Therefore, the sensitivity of the hydrophone at 250 Hz was 20·log_10_(0.180)−153=−167.9 dB re 1 V/µPa. The sensitivity of the measurement hydrophone at 250 Hz determined with a pistonphone calibration was −167.9 dB re 1 V/µPa.

##### In-air relative calibration at 0.25-2 kHz

At low frequencies, hydrophones have the same sensitivity when operated in air as when operated under water. For stimulus frequencies of 1-2 kHz, in-air relative calibration was performed in a smaller un-echoic chamber, with signals emitted through an underwater loudspeaker operating in air (Lubell, LL916). For stimulus frequencies of 125-500 Hz, in-air relative calibration was performed in a classroom using a large aerial loudspeaker. Signals were generated with an Agilent 332220A Arbitrary Waveform Generator. The hydrophone (Reson TC4032, #2112050) was connected to a five-channel amplifier/bandpass filter unit (custom-made by ETEC, Denmark). The received level at the site of the hydrophone was measured with a ½-inch microphone (type 40AF, #95933, with a preamplifier type 26AK, #96888, both from G.R.A.S., Denmark) connected to a power supply (12 AA, G.R.A.S.). The microphone sensitivity (S_M_) had a flat (<1 dB) sensitivity of 50 mV/Pa (or in dB units, S_M_=−146 dB re 1 V/µPa) within the frequency range of 0.1-10 kHz.

The hydrophone and microphone were mounted 5 cm from each other, 1 m from the ground and 2 m from the speaker. The output from both hydrophone and microphone were connected to an Olympus LS10 recorder. The spatial sound field variations in all directions and up to 20 cm range around the hydrophone were measured with the measurement hydrophone to be less than l dB. At low frequencies, the spatial sound field is often surprisingly smooth, even when working in reverberant setups, such as in a classroom.

The hydrophone sensitivity (S_H_) was determined from the equation S_H_=20 log10(V_H_/V_M_)+S_M_, where V_H_ and V_M_ are the voltages from the hydrophone and microphone, respectively (Au and Hastings, 2008).

The sensitivities of measurement hydrophone determined with in-air relative calibration for the frequencies of 125, 250, 500, 1 k and 2 kHz were −167.0, −167.3, −166.4, −167.6 and −168.2 dB re 1 V/µPa, respectively. The sensitivity of the measurement hydrophone obtained with in-air relative calibration was consistently about 3 dB higher than the nominal sensitivity (−170 dB re 1 V/µPa). At 250 Hz, sensitivity determined with in-air relative and pistonphone calibration differed by only 0.6 dB.

##### Reciprocity calibration at 8-64 kHz

The two projectors were spherical HS26 and SP26 hydrophones. Calibrations were made in the acoustic tank at the Marine Biological Research Center, University of Southern Denmark (3 m diameter, 3 m deep) with hydrophones mounted on a triangular PVC rig at 1 m depth with receiver distances of 1.00 m. Sounds were played with a custom-made Labview program from a NI-DAQPAD (USB-6351) to an ETEC PA1001 amplifier and received by an ETEC five-channel amplifier to the NI-DAQPAD and recorded by another custom-made Labview program (both programs courtesy of A. Moriat, National Instruments, Copenhagen, Denmark). At each frequency, 100 pings were played back from two hydrophones and received by the other two. The current driving the playback hydrophone was measured by assessing the voltage over a 5 Ohm resistance connected serially. Calculations of hydrophone sensitivity followed Au and Hastings (2008). For the frequencies of 8, 16, 32, 40, 51 and 64 kHz, the sensitivity determined by the reciprocity calibration was −168.6, −170.8, −170.3, −168.8, −168.3 and −167.6 dB re 1 V/µPa, respectively. All sensitivities were within 1.3 dB of the sensitivities given by the calibration chart supplied by the manufacturer.

##### Averaged and interpolated sensitivities at 250 Hz and 4 kHz

For the frequency of 250 Hz, we estimated sensitivities both by pistonphone calibration and in-air relative calibration to be −167.9 and −167.3 dB re 1 V/µPa, respectively. The sensitivity was determined as the average of those – namely, −167.6 dB 1 V/µPa.

At the frequency of 4 kHz, there was no straightforward way to calibrate with reciprocity or relative calibration techniques. The sensitivity of the hydrophone at 4 kHz was therefore estimated to be the average sensitivity at 2 kHz (−168.2) and 8 kHz (−168.6 dB re 1 V/µPa), or −168.4 dB re 1 V/µPa).

##### Summary of spatial sound field and calibration variations

In Table 3, all compensation to stimulus levels are summarized. The total compensation is the sum of the spatial and sensitivity compensations.

**Table 3.** Compensation for nominal hydrophone sensitivity and spatial sound field variations

Frequency (kHz)	Compensation from spatial sound field variations (dB)	Compensation for nominal hydrophone sensitivity (dB re −170 1 V/µPa)	Total compensation of stimulus levels (dB)
0.125	−1.5	−3	−4.5
0.250	−3.5	−2.7	−6.2
0.500	−1.5	−3.6	−5.1
1	−2.5	−2.4	−4.9
2	+2	−1.8	+0.2
4	−6.5	−1.6	−8.1
8	−8	−1.4	−9.4
16	0	0.8	+0.8
32	0.5	0.3	+0.8
40	2.5	−1.2	+1.3
51	0	−1.7	−1.7
64	−1.5	−2.3	−3.8

As an example of the waveform used for the hearing tests, the 51 kHz stimuli are shown as an oscillogram and spectrogram in Fig. 3.

### Hearing threshold determination

Underwater hearing thresholds were determined from 0.125 to 32 kHz in octave steps and from 32 to 64 kHz in one-third octave steps (Fig. 1). Ten sessions per frequency were conducted, of which ultimately seven to ten sessions could be used to determine the threshold for one frequency before moving on to the next frequency (see Table 1; the number of valid sessions per frequency is given in column ‘*N*’ and the reversals in column ‘Rev.’). The sequence of tested frequencies was 8, 4, 2, 1, 0.5, 0.25, 0.125, 64, 32, 16, 40 and, finally, 51 kHz.

Data from each session had to meet two conditions in order to be included in the threshold determination. All test series had at least four reversals (a switch in the seal's response from a hit to a miss, or from a miss to a hit; Kastelein et al., 2018) and a false alarm rate equal or less than 30%. From the reversals, the mean and s.d. were determined for each session. From these results, the hearing threshold for the animals was determined by calculating the inverse variance-weighted mean and s.d. (Bevington and Robinson, 2003) for each frequency (Table 1).
